# SNP variations in IL10, TNFα and TNFAIP3 genes in patients with dry eye syndrome and Sjogren’s syndrome

**DOI:** 10.1186/s12950-019-0209-z

**Published:** 2019-03-18

**Authors:** Hadas Ben-Eli, Nir Gomel, Doron Jacob Aframian, Rania Abu-Seir, Riki Perlman, Eldad Ben-Chetrit, dror Mevorach, Geffen Kleinstern, Ora Paltiel, Abraham Solomon

**Affiliations:** 1 Braun School of Public Health and Community Medicine, Hadassah-Hebrew University of Jerusalem, POB 12000, 91120 Jerusalem, Israel; 2 Department of Ophthalmology, Hadassah-Hebrew University Medical Center, POB 12000, 91120 Jerusalem, Israel; 3 Department of Optometry and Vision Science, Hadassah Academic College, Jerusalem, Israel; 4 School of Medicine, Hadassah-Hebrew University Medical Center, Jerusalem, Israel; 5 Department of Oral Medicine, Sedation and Maxillofacial Imaging and Sjogren’s Syndrome Center, Hadassah-Hebrew University Medical Center, Jerusalem, Israel; 6 Department of Hematology, Hadassah-Hebrew University Medical Center, Jerusalem, Israel; 7 Unit of Rheumatology, Hadassah-Hebrew University Medical Center, Jerusalem, Israel; 8 Department of Internal Medicine, Hadassah-Hebrew University Medical Center, Jerusalem, Israel; 9 Department of Health Sciences Research, Mayo Clinic, Rochester, MN USA

**Keywords:** Sjogren’s syndrome, Dre eye syndrome, SNP, Anti-inflammatory, Cytokines, Cornea, TNFα, IL-10, TNFAIP3

## Abstract

**Background:**

Cytokines are known to be key players in dry eye syndrome (DES) and Sjogren’s syndrome (SS) pathogenesis. In this study we compared single nucleotide polymorphism (SNP) variations in genes encoding cytokine levels among SS and DES patients in Israel.

**Methods:**

We recruited 180 subjects, 82 with SS and 98 with DES. Using a candidate gene approach and allele-specific PCR technique for genotyping, proportions of risk alleles in Tumor Necrosis Factor α (TNFα) (rs1800629), IinterLeukin-10 (IL-10) (rs1800896) and TNFAIP3 (rs2230926) SNPs were compared between study groups.

**Results:**

Allelic distribution was found very similar to Caucasian (CEU – Utah residents with Northern and Western European roots) population distributions in these SNPs. While none of the SNPs’ variants were significantly associated with SS or DES in a recessive model, in an additive model the TNFα G risk allele was found higher among SS patients compared to DES (Homozygote-G: 84.2% vs. 70.8%; Heterozygote: 26.9% vs. 11.2%, respectively, *p* = 0.02). After adjustment for age, gender and ethnicity, these variants weren’t associated with SS. Genetic scoring reveals that SS patients are more likely to present variants of all three SNPs than DES subjects.

**Conclusions:**

This is the first study evaluating these SNP variations among both patients with DES and patients with SS. We found the allelic distribution in each SNP to be very similar to that found in healthy Caucasian populations presented in the HapMap project. We found the TNFα allele significantly associated with DES for homozygotes, and associated with SS for heterozygotes in the additive model.

## Background

Dry eye syndrome (DES), a chronic inflammation of the ocular surface with potential damage to the external eye, may be one of the clinical manifestations of Sjogren’s syndrome (SS), a systemic Autoimmune Disease (AID) [1, 2]. SS is characterized mainly by severe dry eyes (xerophthalmia) and dry mouth (xerostomia), and can be primary (pSS) or secondary (sSS) to other AID [3, 4]. Both DES and SS are known to be associated with elevated cytokine levels in the serum, including inflammatory cytokines such as IFN-γ, TNFα, IL-1, IL-6, IL-8, IL-12, IL-17 and IL-1β as well as anti-inflammatory cytokines as TGF-β, IL-4 and IL-10 [5–9].

Familial aggregation, candidate gene studies and Genome Wide Association (GWA) studies have suggested a hereditary component in SS establishment [10–13]. However, there is no consensus regarding the role of genes encoding cytokines in the pathogenesis of SS and DES. Several studies have found polymorphisms in genes encoding cytokine levels or other aspects of immune pathways to be associated with SS [10, 12]. Single nucleotide polymorphisms (SNP) in the TNFα and IL-10 genes showed very strong genotype association with pSS subjects in comparison to DES subjects and controls, supporting the hypothesis these genes are main factors in SS immunogenetics [14–16]. However, no association was found in IL10 promoter polymorphism with susceptibility to pSS [17]. The gene TNFAIP3 (rs2230926G) (A20) was found to be highly associated with pSS progression [8, 18, 19].

In this study we wished to compare SNPs’ variations in three genes related to the immune pathway; IL10 (rs1800896), TNFα (rs1800629) and TNFAIP3 (rs2230926), between established SS and DES Jewish patients using a candidate gene approach.

## Methods

### Study population

Recruitment was performed using combined strategies: The first involved approaching SS patients consulting ophthalmology, oral medicine, rheumatology and hematology clinics in Hadassah Medical Center, and DES patients consulting ophthalmology clinics with a complaint of dry eyes. The second recruitment method for DES cases was via an advertisement placed in Hadassah clinics, local newspapers and emails to Hadassah employees, addressing anyone suffering from dry eyes and willing to participate in the study.

Diagnosis of DES was done by a cornea specialist, or by self-reported symptoms. Later, both groups were evaluated with the Schirmer I test (without anesthesia) with score of < 5 mm in 5 min and the Ocular Surface Disease Index (OSDI) questionnaire (with a minimal score > 25) [20] in order to create a common basis for comparison, and also to validate the self-report based diagnoses. The SS diagnosis was based on 4 out of 6 criteria of the US-Euro classification [21] and was done by a rheumatologist, oral surgeon or cornea specialist. Patients fulfilling the inclusion criteria were recruited to the study and DNA was extracted from their peripheral blood using the *salting out* method. Overall 180 participants, 82 with verified SS and 98 with DES, entered the study. All participants signed an informed consent form. Blood samples and data were coded anonymously.

### Genotyping

The polymorphisms of IL10 (rs1800896), TNFα (rs1800629) and TNFAIP3 (rs2230926) were detected using an allele-specific polymerase chain reaction (PCR) assay. DNA was isolated from venous blood, and purified in the “*salting out*” method as described by the Manual Archive Pure DNA Purification Kit (5 Prime, USA). The quality and the quantity of DNA were determined by NanoDrop™ 8000 Spectrophotometric analysis. All samples of SS and DES patients were diluted with Deuterium Depleted Water (DDW) in a final concentration of 10 ng/30 μl. Bi-allelic discrimination was achieved through the competitive binding of two allele-specific forward primers, each with a unique tail sequence that corresponded with two universal fluorescence resonant energy transfer (FRET) cassettes. The assay also contained one reverse primer, and once the reactions were completed and the resulting fluorescence intensity has been measured, the raw data was interpreted to enable genotypes for each DNA sample. For quality control of this assay, inter and intra plating duplicates of about 4% of the samples were sent for genotyping (2 negative controls in each plate), and the result of only one sample of each pair was entered to the final data analysis. Genotyping was performed in “*LGC Genomics*” laboratory in the UK.

### Statistical analysis

Parameters such as age category, gender, ethnicity and the main manifestation of SS disease were presented as proportions. The risk alleles of each SNP were chosen according to current literature and were found to be associated to SS in former studies [14–16, 18, 19]. The prevalence of SNP variations was calculated first in a recessive model, in which the frequency of each allele of the tested SNPs was compared between the two study groups, and then in an additive model, which demonstrated the genotype distribution of homozygotes and heterozygotes for alleles of each SNP in SS and DES groups.

Power calculation of each tested SNP showed that for the given sample size, for the risk allele frequency in Caucasian populations with α = 0.05 and to achieve power (1-β) of 80%, the detectable ORs in the recessive model are: 4.5 for the TNFα, 2.5 for the IL-10 and 6.0 for the TNFAIP3. In the additive model, by the heterozygote frequency, in order to reach power of 80% the detectable ORs are: 2.4, 2.4 and 4.1, respectively.

The comparison of these categorical parameters in the two models was done by a χ^2^ test. A validation of allelic frequencies were performed, using an applicable data from HAP-MAP project, with the expected distributions among CEU (Utah residents with Northern and Western European roots) Caucasian populations [22]. A genetic score was calculated using the sum of SNPs’ polymorphisms, and the relation of this score to the tested disease was analyzed using χ^2^. A logistic regression model, based on additive model and adjusted for confounders, was used in order to demonstrate the OR (odds ratio) for SS compared to DES for each tested SNP. Statistical analysis was done using SPSS 23.0 software (Chicago, IL 60606–6307), with α = 0.05, and power calculation was done using WinPepi 11.63 software.

## Results

A total of 180 patients entered this study, with a mean age [and SD] of 56.7 [13.1] years for the SS group and 50.0 [15.2] for the DES group and overall age range of 19–86 (Table 1). As expected, most of the study participants were women in both the SS and the DES groups (*N* = 75; 91.5% and *N* = 68; 69.3%, respectively). Eastern European ancestry was the most common ethnicity (39% in SS and 56.1% in DES). The main clinical manifestations among SS patients had a fairly even distribution, the chief complaint being dry eyes among 26.6% of patients, dry mouth among 34.2%, and joint pain among 39.1% (Table 1). Although division to SS subgroups according to disease’ manifestations was presented, aa comparison of the allelic distribution of these subgroups to DES group was not performed due to a relatively small numbers of patients with different allelic distribution within these subgroups. Therefore, the genotyping analysis considered all patients with SS as one group.

**Table 1:** Characteristics of study population

	SS^*^ (*n* = 82)	DES^†^ (*n* = 98)
Mean age [SD^‡^]	56.7 [13.1]	50.0 [15.2]
Age range	20–86	19–83
Female gender: n (%)	75 (91.5)	68 (69.3)
Ethnicity: n (%)		
Eastern Europe	32 (39)	55 (56.1)
West Asia	20 (24.4)	16 (16.3)
North Africa	19 (23.1)	10 (10.2)
Israel	6 (7.3)	8 (8.1)
Mixed	5 (6.1)	9 (9.2)
SS manifestation: n (%)		
Eyes	22 (26.6)	NA
Mouth	28 (34.2)	NA
Joints	32 (39.1)	NA

* - ss (Sjogren’s Syndrome); † − DES (Dry Eye Syndrome); ‡ - SD (standard deviation)

Genotyping tests produced identical results for all duplicate samples. Table 2 lists the frequencies of the various alleles in a recessive model, in which the risk allele was counted if it was present either once or twice in the same individual in the genotype for each SNP. The frequency of having two risk alleles in the tested SNPs was found to be higher in SS group compared to DES, but the difference was not significant. Risk allele G in SNPs rs1800629 was found to be more frequent among SS patients (84.2%) compared to DES patients (70.3%) (*P* = 0.56). In rs1800896-A the frequency of the risk allele was 62.1% in SS and 57.2% in DES (*P* = 0.23), and in rs2230926-G the frequency of risk allele was lower in the SS group (5.9%) than the DES (8.4%) (*P* = 0.54). When comparing these alleles’ frequencies distribution in the recessive model to a control CEU (Caucasian) population, as presented in the International HAP-MAP project, only minor differences were noted.

**Table 2:** Distribution of SNP variations among study participants - Additive model

SNP	Risk Allele		SS^a^ (n = 82) n (%)	DES^b^ (n = 98) n (%)	P (χ^2^)	CEU^c^ Freq. (HapMap)	
*TNFα (rs1800629)*	G	Homozygote G	58 (70.8)	83 (84.7)		67.3	
		Heterozygote	22 (26.9)	11 (11.2)		31	
		Homozygote A	1 (1.3)	2 (2.1)		1.8	
					**0.02**		
*IL10 (rs1800896)*	A	Homozygote G	13 (15.9)	21 (21.4)		27.4	
		Heterozygote	36 (44.0)	42 (42.9)		51.3	
		Homozygote A	32 (39.1)	35 (35.7)		21.2	
					0.64		
*TNFAIP3 (rs2230926)*	G	Homozygote T	73 (89.2)	85 (86.7)		94.7	
		Heterozygote	8 (9.8)	10 (10.3)		5.3	
		Homozygote G	0	0		0	
					0.54		

^a^- ss (Sjogren’s Syndrome); ^b^ − DES (Dry Eye Syndrome)

^c^ − CEU (C): Utah residents with Northern and Western European ancestry from the CEPH collection. Data is available at Hapmap

Table 3 demonstrates the polymorphism in an additive model, giving greater weight to the risk allele homozygote relative to the heterozygote or homozygote of the other allele. The rs1800629-G allele was found to be significantly associated with DES compared to SS for homozygotes of the risk allele (84.7 and 70.8% respectively) (*P* = 0.02). The frequency of homozygote and heterozygote rs1800896-A risk allele was higher in SS patients (39.1 and 44.0% respectively) than DES patients (35.7 and 42.9% respectively), but the difference was not statically significant (*P* = 0.64). The SNP variation distribution was found to be similar for the rs2230926-G allele among SS and DES for homozygote and for heterozygote to the risk allele. When comparing these alleles’ frequencies distribution in the additive model to a control CEU (Caucasian) population, as presented in the International HAP-MAP project, only minor differences were noted in all tested SNPs.

**Table 3:** Distribution of SNP variations among study participants - Recessive model

SNP	Risk Allele	Allele	SS^a^ (n = 82) %	DES^b^ (n = 98) %	P (χ^2^)	CEU^c^ Freq. (HapMap)
*TNFα (rs1800629)*	G					
		G Allele	84.2	70.3		82.7
		A Allele	15.8	29.7		17.3
					0.56	
*IL10 (rs1800896)*	A					
		G Allele	37.9	42.8		53.1
		A Allele	62.1	57.2		46.9
					0.23	
*TNFAIP3 (rs2230926)*	G					
		T Allele	94.1	91.6		97.3
		G Allele	5.9	8.4		2.7
					0.54	

^a^ - ss (Sjogren’s Syndrome); ^b^ − DES (Dry Eye Syndrome)

^c^ − CEU (C): Utah residents with Northern and Western European ancestry from the CEPH collection. Data is available at Hapmap

A genetic score (Table 4) was constructed by summing the prevalence of 1, 2 or 3 SNPs’ variations in each individual with SS or DES. These results show that more patients with DES carry 1 or 2 of the risk alleles of the tested SNPs than SS patients (58.3% vs 41.7 and 54.5% vs 45.5%, respectively), while variation in three SNPs was more common in SS than DES (58.3% vs 41.7), but the findings were not statistically significant (*P* = 0.60). Furthermore, no association was found among these three tested SNP’s in genetic scoring.

**Table 4:** Genetic scoring for SNP variations

SNP	SS^a^ (*n* = 82) n (%)	DES^b^ (*n* = 98) n (%)	Total n (%)	P (χ^2^)
1 SNP	15 (41.7)	21 (58.3)	36 (100)	
2 SNP	60 (45.5)	72 (54.5)	132 (100)	
3 SNP	7 (58.3)	5 (41.7)	12 (100)	0.60

^a^ - ss (Sjogren’s Syndrome); ^b^ − DES (Dry Eye Syndrome)

The logistic regression model (Table 5) revealed that SS is associated with female gender (OR = 3.62; 95% CI: 1.36–9.62), younger age (OR = 0.96; 95% CI: 0.93–0.98) and Eastern Europe ethnicity (OR = 0.39; 95% CI: 0.19–0.78 vs other). None of the three tested SNPs was found to be associated with SS in the logistic regression model.

**Table 5:** Association with Sjogren’s syndrome using logistic regression model

Variable	OR^a^	95% CI^b^
Age	0.96	**0.93–0.98**
Gender	3.62	**1.36–9.62**
Ethnicity (Eastern Europe vs. other)	0.39	**0.19–0.78**
TNFα(rs1800629)-G	0.86	0.04–17.5
IL10 (rs1800896)-A	1.06	0.50–2.24
TNFAIP3 (rs2230926)-G	1.02	0.33–3.15

^a^ - OR = Odds ratio; ^b^ − CI = Confidence interval

## Discussion

Our study is the first to evaluate all these three SNP variations among both patients with DES and patients with SS. We found the allelic distribution in each SNP among patients with SS and DES very similar to that found in Caucasian populations (CEU) presented in the HapMap project, which validates our findings. The TNFα(rs1800629-G) allele was found to be significantly associated with DES for homozygotes, and associates with SS for heterozygotes in the additive model. Our study reveals, using genetic scoring, that SS patients are more likely to have all these three SNPs’ variations than DES patients. Furthermore, females were found to have a higher genetic risk for SS compared to DES.

Several studies have found an association between SS and polymorphisms in genes encoding cytokines TNFα (rs1800629), IL10 (rs1800896) and TNFAIP3 (rs2230926), but have not found correlation to DES [14–16, 18]. Yet, in other works, this association was not shown [17, 23]. In our study we compared the allelic frequency of the risk allele in these three SNPs among SS patients vs. those with DES. The allelic distribution in each SNP was very similar to that found in Caucasian populations (CEU). We found that only the TNFα (rs1800629-G) allele was significantly associated with DES for homozygotes, and associates with SS for heterozygotes in the additive model. The TNFα gene promoter is known to be associated with TNFα protein levels, inflammation, outcome of infection, susceptibility to autoimmune diseases such as SS, and also with the most serious complication associated with SS, i.e. non-Hodgkin lymphoma [14].

The results of the genetic scoring demonstrate that individuals with all three SNP variations tested in this study have a higher likelihood of having SS than DES. As expected, female gender has the strongest effect on the risk of SS, while Eastern European ethnicity had a protective effect on the disease. When adjusting for possible confounders, these SNPs were not found to be risk factors for SS.

Our study was limited by a small sample size which resulted in limited power, and also by the fact that the comparison was only between SS and DES subjects, who may have a similar immune system hyperactivity characteristics. Additionally, the study did not include a healthy control group.

Using a candidate gene approach, IL10 (rs1800896-A) and TNFAIP3 (rs2230926-G) were not found to be significantly more strongly associated with SS compared to DES. The TNFα (rs1800629-G) SNP was found to be associated with SS and not with DES in an additive model. These results emphasize the need for further studies comparing between SS, DES and healthy controls, and studies on larger populations and different ethnic groups. Further research should include healthy control subjects, a larger number of subjects within SS subgroups and should analyze protein levels for the three cytokines in the patients’ serum. Moreover, further exploration of other SNPs’ variations related to the immune pathway, which may play a role in SS establishment, is needed. SS is a non-common disease and this study demonstrates the importance of consortia and collaborative studies in order to study genetic susceptibility to autoimmune diseases.

## Conclusions

In this study we explored the association between three SNP’s of genes encoding cytokines and relate to the immune pathway and between common disease, DES, as well as SS. The allelic distribution in each SNP was found to be very similar to that found in healthy Caucasian populations presented in the HapMap project. TNFα allele was found to be significantly associates with DES for homozygotes, and associates with SS for heterozygotes in the additive model. The IL-10 allele and TNFAIP3 allele weren’t found to be significantly associated with SS nor DES. Further research will help revealing additional alleles that can be associated with these syndromes leading to a better understanding it’s immune pathway.

## Abbreviations

AID: Autoimmune disease
CI: Confidence interval
DDW: Deuterium depleted water
DES: Dry eye syndrome
FRET: Fluorescence resonant energy transfer
GWA: Genome Wide Association
IL-10: IinterLeukin-10
OR: Odds ratio
OSDI: Ocular surface disease index
PCR: Polymerase chain reaction
SNP: Single nucleotide polymorphism
SS: Sjogren’s syndrome
TNFα: Tumor Necrosis Factor α
